# Mobile Application for Promoting Gluten-Free Diet Self-Management in Adolescents with Celiac Disease: Proof-of-Concept Study

**DOI:** 10.3390/nu13051401

**Published:** 2021-04-21

**Authors:** Sonya Meyer, Gali Naveh

**Affiliations:** 1 Department of Occupational Therapy, Ariel University, Ariel 40700, Israel; 2 Department of Industrial Engineering and Management, Shamoon College of Engineering, Beer-Sheva 8410802, Israel; galinaveh@sce.ac.il

**Keywords:** celiac, self-management, mobile application, system usability, user satisfaction

## Abstract

Celiac disease (CD) is a chronic disease treated by maintaining and managing a lifelong restrictive gluten-free diet. The purpose of this study was to develop a mobile application, Plan My C-Day, to promote self-management skills among youth with CD during adolescence—a time when decreased adherence often occurs—and examine its usability among adolescents with CD. Plan My C-Day contains three simulations of activities involving eating out and actions to take when preparing for these events. It was developed and pilot tested by 13 adolescents with CD. Application use and user perception data were collected and analyzed. Participants chose 160 actions within the simulations. For over 75% of participants, the time to complete the simulation decreased from the first to the third (last) simulation by an average of 50%. The average reported usability perception was 3.71 on a scale of 1 to 5, with system ease of use and ease of learning obtaining the highest scores. This study demonstrated that the Plan My C-Day mobile application’s self-management content, features, and functions operated well and that the simulations were easy to understand and complete. Further development will include the option to add self-created activities and adaptation to different languages and cultures.

## 1. Introduction

Celiac disease (CD) is a chronic disease triggered by exposure to gluten, a protein found in foods such as wheat, barley, and rye, in people with genetic predisposition to the disease [1]. The currently known prevalence is approximately 1% of the population, and it is one of the most common chronic disorders in childhood [1,2,3]. The only treatment available to date is complete avoidance of gluten consumption and lifelong adherence to this strict gluten-free diet [2,4].

Disease management is a term in health care that expresses the continuous management of chronic conditions over time and the process of people accepting long-term responsibility for themselves and their health conditions [5,6]. Self-management is the interaction of health behaviors and related processes with the lifetime tasks that people engage in to care for themselves and live well with a chronic condition [7,8]. Self-management is a broader concept compared to adherence because it emphasizes the person’s active role in the decision-making process [9]. Self-management consists of an array of skills, such as decision-making, carrying out plans, being flexible, and solving problems to support learning, remembering, planning, and deciding [10,11]. Effective self-management can be achieved by creating health patterns at an early age, providing strategies for disease prevention and management throughout life, and building the ability to navigate daily challenges and solve problems [12,13]. People with chronic health conditions are responsible for managing their health, including maintaining daily needs and adhering to changes in their lifestyle [14]. Specifically, self-management skills significantly contribute to maintaining a proper dietary regime [15]. Moreover, self-management of health and disease has been recognized as a topic of importance and relevance by the European Commission [16].

When children transition to adolescence, they strive for autonomy from parental control, and adolescents become experts in their self-care [17]. Adolescents with chronic health conditions such as CD become more involved in making health-related decisions as they mature and gradually develop the skills needed to self-manage their health conditions [18,19]. Thus, promoting effective skills to self-manage a gluten-free diet to maintain health is particularly important in the transitional period of adolescence—when a decrease in the rate of adherence often occurs [3]. The knowledge, skills, and confidence for self-management are tools acquired not through education alone. Attaining these abilities requires cognitive skills and transition planning that includes developing independence in self-management tasks [15,20,21]. Promoting effectiveness in carrying out a task requires understanding of the cognitive strategies used by the person to accomplish the task and understanding how to develop these strategies [22]. A cardinal constituent of the strategy-acquiring process is the person’s self-awareness of performance. This reflects the relationship between knowledge, beliefs, the particular task’s demands, and the specific context of the task to identify errors or less effective strategies and correct them both during and after completing the task [23]. These strategies and skills are vital when coping with new challenges in daily life, and relationships between such cognitive skills and health behaviors while taking part in various food-related activities among adolescents with CD have been found [21].

In recent years, the number of mobile applications related to health and disease management among adolescents has increased. These include applications for adolescents with chronic conditions, such as diabetes, inflammatory bowel disease, and cystic fibrosis [24,25]. Mobile health (mHealth) is a way to promote health by applying mobile technologies to improve health outcomes. They are cost-effective, convenient, easily accessible, personalizable, and can be operated without professional health care assistance [26,27]. mHealth-based approaches have become very popular in chronic health care management and include, among other things, automated text reminders, frequent and accurate symptom monitoring, daily reminders for medication use, follow-up of food intake, education, tips, support, and condition tracking [25,28,29]. As such, technological interventions have the potential to contribute to managing gastrointestinal health conditions such as CD [30].

Applications, not specifically defined as medical devices, that are dedicated to CD or gluten-free diets are available in many countries and languages. They are often offered by celiac organizations and other initiatives, mostly to provide information about gluten-free diets, recipes, products, stores, and restaurants. Applications that specialize in CD have been continuously developed in recent years. For example, the Text Message Intervention (TEACH) [31] was developed as an intervention approach for adolescents with CD to promote dietary adherence, patient activation, and quality of life. MyHealthyGut (KORE Digital Health Therapeutics Inc., Vancouver, BC, Canada, available from the Apple store) was developed for adults with CD or gluten intolerance [15] to improve effective management. This application focuses on self-management strategies such as diet tracking, symptom journaling, meal-plan content, education, supplements, and recommended foods [32]. More recently, an application aiming to serve as a platform for interactions between people with CD and their social circles was developed to exchange celiac-related information and create a safe environment in which people with CD can socialize safely [33].

However, to date, no application has been found that focuses specifically on developing self-tailored self-management cognitive skills (e.g., decision-making, plan execution, flexibility, and problem-solving) for adolescents with CD while maintaining their strict gluten-free diets and participating in their daily activities [11,15,34]. Hence, we proposed an application that focuses on the planning stage before taking part in food-related activities that adolescents encounter in their daily lives inside and outside of their home environment, based on the previously published Celiac Disease Children’s Activities Report (CD–Chart) [34]. Contrary to existing applications, the focus of this application is on planning and choosing the actual actions that the adolescents need to take before the event to successfully manage and maintain the diet while participating in the event and self-rating the created plan.

Therefore, the objectives of this study were to: (1) design and develop the theoretically based “Plan My C-Day” smartphone application to promote self-management among adolescents with CD, (2) pilot test the use of the Plan My C-Day application, and (3) gather feedback from end users concerning how they perceive the contribution of the application to their self-management, as well as the application’s usability and their general satisfaction from it, to determine its potential and plan further development. It is hypothesized that a significant difference will be found between the user time on the simulations, the functionality of the mobile application will be relevant to adolescents with CD, and users will report that the application is easy to use and has potential to contribute to their daily health management. This is a proof-of-concept study that describes the beginning stages of the new application’s development [35], aimed at estimating the potential of the “Plan My C-Day” application. The results from this pilot study will be used to improve the app itself and continue its development.

## 2. Materials and Methods

### 2.1. Study Design, Participants and Procedure

This proof-of-concept study consisted of a trial use of the mobile application by users. An online questionnaire was used to investigate participants’ user perceptions following trial use. The study was approved by the Human Subjects Research Committee of the Department of Industrial Engineering and Management at Ben-Gurion University of the Negev (protocol code 2020/12, date of approval 19 February 2020).

Thirteen adolescents with CD, aged 13 to 18 years (*M*_age_ = 15 years, 76.9% female), participated in pilot testing Plan My C-Day. Based on self-reports, participants were diagnosed with CD between 1 and 13 years prior to participating in the study (*M* = 8.15 years, *Mdn* = 11.0). Inclusion criteria were adolescents aged 13 to 18 years diagnosed with CD, fluent in the Hebrew language, and possessing an Android smartphone. Users for the pilot test of the application were recruited via celiac social media groups in Israel during the first week of May 2020.

Potential participants who responded to the recruitment advertisements were provided with explanations and instructions about the study, and the adolescents and their parents signed written consent forms. The adolescents were then given the link to the application on the app store, a username, and a password. Clear instructions concerning installing and using the application, and completing the online questionnaire, were provided to ensure equal exposure and knowledge about the application and to avoid bias. Users were instructed to explore the application and follow the instructions. Upon completing the three-simulation trial use of the application, all users were sent a link to an online survey to obtain their user perception feedback. Participant data were collected between 10 May and 18 May 2020, and no follow-up was conducted after data collection.

### 2.2. Mobile Application

**Figure 1:**
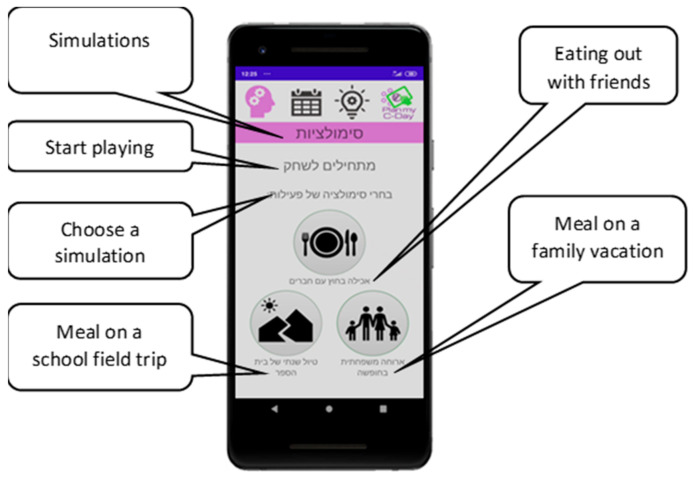
Three “Plan My C-Day” simulations.

The application, “Plan My C-Day,” was developed using Android Studio and Firebase as a cloud-based database by a group of undergraduate students, as part of their program capstone project. It is available (on Google Play) for smartphones with Android operating systems. It requires logging in with a username and password generated by the system administrator (the researchers). The main functionality of this first version of the application is the simulation of preparing oneself to participate in away-from-home food-related events. Three food-related events were chosen for this purpose based on the event included in the previously developed CD Chart questionnaire [34]: eating out with friends, a meal on a family vacation, and a meal during a school fieldtrip (Figure 1).

After choosing one of the three simulation activities, the user is invited to select from a list of actions that they would plan to do to participate in the upcoming activity while maintaining a gluten-free diet. These actions were previously generated by children and adolescents with CD when interviewed by the first author as part of the CD Chart development [34]. For this study, the researchers selected 66 optional actions that users could perform before participating in the food-related activity, and these actions were organized into eight categories to make finding and choosing them more accessible, based on the main action characteristics: ask, prepare, check, avoid, take, collaborate, call, and say (Table 1).

**Figure 2:**
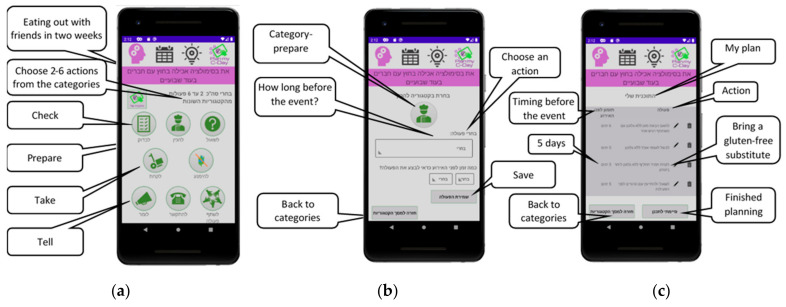
Simulating preparation for an event: (**a**) choosing category of actions, (**b**) choosing an action and scheduling, and (**c**) self-created plan.

The categories are visually represented by icons (Figure 2a). Next, the user is asked to schedule when he or she should perform that action by choosing the number of hours, days, or weeks prior to the activity (e.g., call the restaurant to check the gluten-free option on the menu a week prior to an arrangement to eat out with friends) (Figure 2b). The user is asked to choose between two and six actions to create their personalized plan. This plan can then be edited and adjusted prior to finalizing the plan (Figure 2c).

**Figure 3:**
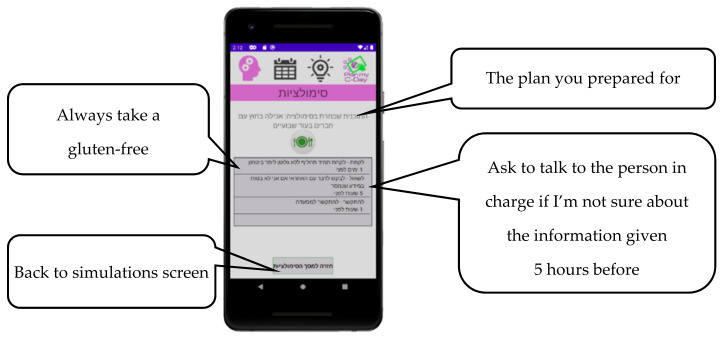
Finalized self-created plan for preparing for eating out with friends.

After users confirm their plan, they are asked to self-rate their self-created plan on a five-star scale, specifically rating their own performance and evaluating the effectiveness of their choices [23]. A sample of a finalized plan is shown in Figure 3.

### 2.3. Application Use and User Perception

Two aspects were explored in this pilot study: use of the application and users’ perceptions of it. Use was examined based on the time that the users spent in the application and in each simulation; their behavior in the application—specifically, the number and diversity of actions chosen from the categories—and their rating of their plans. User perception was obtained using a questionnaire that focused on five aspects: (i) self-management, (ii) usefulness, (iii) ease of use, (iv) ease of learning, and (v) general satisfaction. The first part focused on the contribution of the application to the users’ ability to manage their dietary needs in the various food-related events. It comprised seven CD self-management -specific items phrased for this study by the authors, based on the theoretical background of the application (e.g., “Using the application helped me learn to plan what to do in preparation for participating in activities involving food,” and “Using the application helped me learn to plan when to do different activities in preparation for participating in activities involving food”). The remaining four parts were based on the widely used and previously validated tool in the field of technological systems evaluation, the Usefulness, Satisfaction, and Ease of Use Questionnaire (USE) [36]. Respondents were asked to indicate their agreement with 34 statements on a 5-point Likert-scale from 1 (strongly disagree) to 5 (strongly agree). For example, one item asked the respondents to indicate how strongly they agreed with the statement, “The application is user friendly, and I quickly became skillful with it.” Scores showed acceptable levels of internal reliability for each of the five user perception aspects (Cronbach’s alpha ranged from α = 0.81 to α = 0.95).

### 2.4. Data Analysis

Data were analyzed with IBM SPSS Statistics Version 26 (IBM, Armonk, NY, USA). Means and standard deviations (SD) were calculated to describe the sample demographic and application use characteristics (i.e., time spent in the application, time spent on each simulation, the number and diversity of actions chosen from the different action categories, and self-rating of plans). The internal reliability of the user perception questionnaire was determined with Cronbach’s α. Performance time in each of the three simulations was assessed by within-subjects repeated-measures ANOVA.

## 3. Results

### 3.1. Application Use

**Figure 4:**
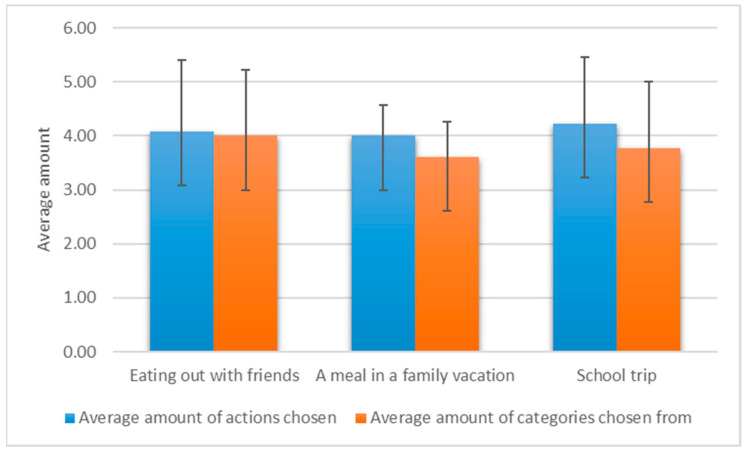
Average and standard deviation of number of actions chosen and categories chosen from per simulation per user.

**Figure 5:**
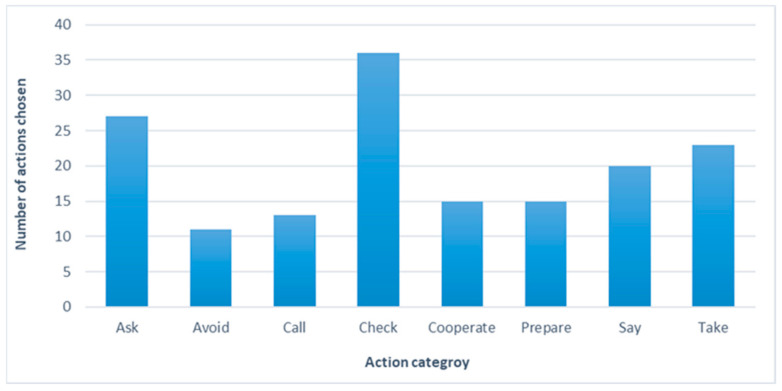
Number of actions chosen from each category.

The average overall time that users spent in the application was 16:23 min (SD = 6:49), with a mean time of 3:33 min on each simulation. For 76.92% of the users, the time to complete the simulation decreased from the first simulation to the last (third) by an average of 50%. Two (15%) users spent almost the same amount of time (difference < 20 s) in the first, second, and third simulation activities. Only one (7.5%) user showed a significant difference between time spent on the first (1:44 min) and third (3:18 min) simulations. Mauchly’s test indicated that the assumption of sphericity had been violated, *χ*^2^(2) = 15.29, *p* < 0.05; therefore, degrees of freedom were corrected using Greenhouse–Geisser estimates of sphericity (ɛ = 0.57). The results show a significant difference between the time spent on the three simulations, *F* (1.1, 13.71) = 4.64, *p* = 0.04, η^2^ = 0.279. Users selected an average of 4.1 actions for each simulation, from 3.8 categories (Figure 4). This resulted in a total of 160 actions selected in all three simulation plans, ranging from 13 to 36 actions from each of the eight categories chosen by each user (Figure 5).

From each of the eight categories, some actions (e.g., asking at the event what does or does not contain gluten or calling the restaurant) were chosen by most participants, whereas other actions were chosen by only one user (e.g., notify my parents about the event and tell myself I am coming to have fun and not to eat). Fifty percent of the actions were chosen more than once. Additionally, four (30%) users made changes to their choices prior to final confirmation of the plan. The users’ self-rated levels of satisfaction with their plans ranged from three to five stars.

### 3.2. User Perception

**Figure 6:**
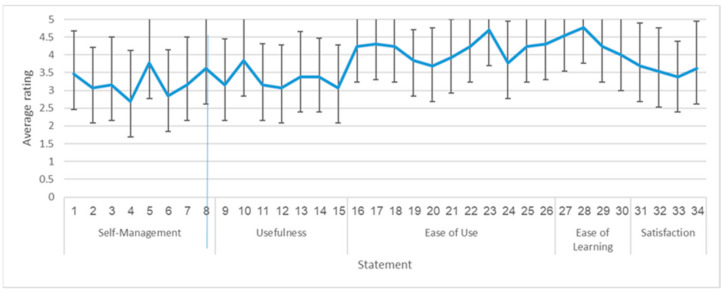
Average and standard deviation of rating of statements on user perception based on the Ease of Use Questionnaire (USE) [36] (see Section 2.3.) (*N* = 13, self-rated on 5-point Likert scale between 1 (strongly disagree) and 5 (strongly agree)).

The average total score of the user perception items was 3.71, with scores ranging from 2.71 to 4.68 (Mdn 3.82) on the 34 items. The lowest ratings were assigned to questions dealing with the application’s contribution to the users’ self-management of their dietary limitation, whereas the highest rankings were assigned to system ease of use and ease of learning (Figure 6).

In addition, some of the users initiated sharing their impressions with the authors or on social media where they were recruited by sending text messages after completing the application. For example, “I tried out the App. It’s great. I recommend you sign up too”, “A cool App”, and “An excellent application”.

## 4. Discussion

Plan My C-Day is a mobile application developed specifically to promote self-management skills among adolescents with CD. The purpose of this study was to create and pilot test the Plan My C-Day application and to examine its usability among adolescent users with CD. The creation of this application was theoretically based on the cognitive components required in self-management and findings from previous research involving adolescents with CD [11,34]. User behavior while using the application was collected, as was users’ feedback regarding their perceptions of the application’s usability and contribution to self-management.

Overall, the time that the users spent on the application—including exploring, understanding instructions, and performing the simulations—was around 15 min; less time was needed as they proceeded through the three simulations. These results may indicate that the application was easy to learn for most users and that they acquired and used the information adaptively [37]. Furthermore, their active-practice learning process progressed and improved as they used the application and advanced through its various simulations and changing demands [38].

Results showed that users chose from all the possible actions divided into the eight action categories provided in the application. Some actions were used more commonly, whereas others were used only once. This may highlight the importance of having a variety of actions of choice to enable each user to self-create a plan most suitable to their own coping style and health management behavior [11,18]. Nevertheless, that users chose half of the actions more than once reinforces the suitability of the actions provided in the application. As previously mentioned, this array of choices was based on actions originally generated by children and adolescents with CD when interviewed during the development of the CD Chart questionnaire [34].

The changes performed by some users before finalizing their self-created plans, as well as the variety and range of self-reported satisfaction with their plans, may reflect the users’ attentiveness while using the application. Indeed, processes such as checking, changing, and decision-making are all cognitive skills involved in self-management and required when managing CD [10,11,21].

User perception questions related to the contribution of the application to users’ dietary self-management obtained lower ratings compared to user perception questions regarding other areas. This may be due to the current application’s limited functionality (i.e., simulation only). Additionally, given the sample’s characteristics—mostly adolescents with many years of experience in conducting themselves in these situations—the application may have seemed less of a need in these simulated events. Nonetheless, these participants would possibly find more of a contribution if the application provided additional event options. The user perception questions with the highest scores on the questionnaire related to ease of use and ease of learning. Because ease of use (together with usefulness) is identified as a strong determinant of technology adoption [39] and ease of learning is considered a cornerstone of usability [40], these findings suggest that the Plan My C-Day application design supports a relatively simple adoption by users.

The results of this study should be interpreted with caution considering the limitations of the study. First, the sample size was small, and the application was developed in the Hebrew language; thus, its generalizability is limited. In addition, because our principal goal was the proof-of-concept exploratory phase of the newly developed application, we have not yet generated clinically meaningful or follow-up data. Furthermore, because, to the best of our knowledge, no similar application has been developed and tested in the past, we could not collate our results with previous research. Therefore, generalization of the results may be limited. Secondly, due to resource limitations, the application was designed at this stage for only the Android platform. Nevertheless, the aim of this proof-of-concept study was to examine the application’s basic concept as an initial research stage. In further research, we will conduct feasibility and efficacy testing with larger samples and clinically relevant data.

## 5. Conclusions

Effective self-management skills for a gluten-free diet in CD, which are vital to managing the health condition, involve navigating daily challenges, solving problems, and making decisions [9,12,13]. Learning, practicing, and acquiring these skills is particularly important in the crucial developmental–transitional period of adolescence [3]. In recent years, mHealth has been found to be useful in promoting chronic health management [25]. This proof-of-concept study demonstrated that the content, features, and functions of the Plan My C-Day mobile application operated well, and the simulations were easy to understand and complete. Plan My C-Day is a mobile application designed specifically to promote self-management cognitive skills among adolescents with CD and shows promise as a baseline to progress with further development. Further development should create the option to add self-created activities tailored to each individual’s daily life events and to adapt the simulations to different languages and cultures.

## Figures and Tables

**Table 1:** “Plan My C-Day” 66 actions and eight action categories.

Action Category	Actions
ask	Ask at the place what is/isn’t gluten-free
	Ask someone else to ask for me
	Ask the person who prepared the food, what are the ingredients
	Ask questions before so as not to be disappointed
	Ask who the manufacturer of the product is
	Ask to speak to the person in charge if not sure about the information provided
	Ask what’s the plan, before the event
	Ask someone to prepare GF substitutes for me before the food activity
	Ask who to contact to get the gluten-free serving
	Ask before the event if there are any gluten-free accommodations
avoid	Remind myself to avoid anything that says “may contain gluten” in the ingredients
	Remind myself to strictly adhere to a gluten-free diet
	Remind myself to check even when seeing someone else with celiac disease eating something
	Remind myself to only drink if there are no gluten-free options
	Remind myself to maintain self-discipline
	Remind myself that if unsure—do not eat
	Remind myself to avoid food I am not familiar with
	Tell myself I came to enjoy myself and not to eat
take	Bring myself a gluten-free substitute
	Keep a box with substitutes at the place of the activity
	Always take a gluten-free substitute for just in case
	Take basic gluten-free products
	Take equipment for preparing gluten-free food
	Take gluten-free food in a cooler bag
	Take a wallet with money in case something is missing
	Buy gluten-free products
check	Check information online, on social networks
	Check before a vacation how to say “gluten” in the local language
	Check what needs to be brought from home in advance
	Make sure the information on the Internet is up-to-date and accurate
	Check and make sure before eating a dish that is defined as gluten-free
	Check the packaging of refreshments/food distributed
	Check in advance what is safe/not safe in order to be prepared accordingly
	Check if the menu includes gluten-free dishes
	Check in advance what will be available, don’t take a chance
collaborate	Ask and consult with parents before an activity
	Coordinate bringing gluten-free food with another sensitive participant
	Prepare gluten-free food with someone else
	Talk to friends about finding places with gluten-free options
	Keep up-to-date with friends when planning to bring refreshments
	Take part in planning a social gathering
	Take interest in the menu
make	Take responsibility
	Cook gluten-free food for myself
	Prepare myself reminder notes
	Send myself cell phone reminders
	Prepare in advance
	Make sure to eat before an event with food
	Bake gluten-free cakes/cookies
phone	Call the restaurant
	Call a friend before visiting
	Call and find out if it is appropriate to go to a place that has gluten-free options
	Call the person in charge to find out about gluten-free options
	Call to ask for gluten-free substitutes
	Call parents to double check
	Call the venue to find out about gluten-free preparation
tell	Update parents about an event or activity information
	Ask the waiter at the restaurant for a gluten-free menu
	Tell the person in charge of the event that I have celiac disease
	Mention before dinner that I should be first
	Inform friends
	Say at the event that I need a gluten-free dish
	Tell others what I can and can’t eat
	Inform relevant parties
	Remind people so not to fall between the cracks
	Tell the person in charge that I want to see packaging

## Data Availability

The data presented in this study are available on request from the corresponding author. The data are not publicly available due to ethical restriction.
